# The miR-20a/miR-92b Profile Is Associated with Circulating γδ T-Cell Perturbations in Mild Psoriasis

**DOI:** 10.3390/ijms24054323

**Published:** 2023-02-21

**Authors:** Stana Tokić, Maja Jirouš, Vera Plužarić, Martina Mihalj, Marija Šola, Maja Tolušić Levak, Kristina Glavaš, Peter Balogh, Mario Štefanić

**Affiliations:** 1 Department of Laboratory Medicine and Pharmacy, Faculty of Medicine, University of Osijek, 31000 Osijek, Croatia; 2 Department of Medical Chemistry, Biochemistry and Clinical Chemistry, Faculty of Medicine, University of Osijek, 31000 Osijek, Croatia; 3 Department of Dermatology and Venereology, University Hospital Osijek, 31000 Osijek, Croatia; 4 Department of Physiology and Immunology, Faculty of Medicine, University of Osijek, 31000 Osijek, Croatia; 5 Department of Histology and Embryology, Faculty of Medicine, University of Osijek, 31000 Osijek, Croatia; 6 Department of Transfusion Medicine, Faculty of Medicine, University of Osijek, 31000 Osijek, Croatia; 7 Department of Immunology and Biotechnology, Faculty of Medicine, University of Pecs, 7622 Pecs, Hungary; 8 Department of Nuclear Medicine and Oncology, Faculty of Medicine, University of Osijek, 31000 Osijek, Croatia

**Keywords:** hsa-mir-20a, hsa-mir-92b, hsa-mir-29a, hsa-let-7c, psoriasis vulgaris, γδ T cells

## Abstract

Psoriasis vulgaris (PV) is an autoinflammatory dermatosis of unknown etiology. Current evidence suggests a pathogenic role of γδT cells, but the growing complexity of this population has made the offending subset difficult to pinpoint. The work on γδTCR^int^ and γδTCR^hi^ subsets, which express intermediate and high levels of γδTCR at their surface, respectively, is particularly scarce, leaving their inner workings in PV essentially unresolved. We have shown here that the γδTCR^int^/γδTCR^hi^ cell composition and their transcriptom are related to the differential miRNA expression by performing a targeted miRNA and mRNA quantification (RT-qPCR) in multiplexed, flow-sorted γδ blood T cells from healthy controls (*n* = 14) and patients with PV (*n* = 13). A significant loss of miR-20a in bulk γδT cells (~fourfold decrease, PV vs. controls) largely mirrored increasing Vδ1^-^Vδ2^-^ and γδ^int^Vδ1^-^Vδ2^-^ cell densities in the bloodstream, culminating in a relative excess of γδ^int^Vδ1^-^Vδ2^-^ cells for PV. Transcripts encoding DNA-binding factors (*ZBTB16*), cytokine receptors (*IL18R1*), and cell adhesion molecules (*SELPLG*) were depleted in the process, closely tracking miR-20a availability in bulk γδ T-cell RNA. Compared to controls, PV was also associated with enhanced miR-92b expression (~13-fold) in bulk γδT cells that lacked association with the γδT cell composition. The miR-29a and let-7c expressions remained unaltered in case–control comparisons. Overall, our data expand the current landscape of the peripheral γδT cell composition, underlining changes in its mRNA/miRNA transcriptional circuits that may inform PV pathogenesis.

## 1. Introduction

Psoriasis vulgaris (PV) is a debilitating autoimmune dermatosis with a complex etiology and lifelong duration. Psoriatic arthritis, diabetes, and cardiovascular disorders often accompany the skin manifestations, making PV a systemic and highly polymorphic condition [1,2,3,4,5]. Currently, PV is considered a T-cell-driven disease, and conventional αβ T cells have been assigned a major role in epithelial, stromal, and vascular skin remodeling. Emerging evidence, however, demonstrates that innate-like lymphocytes, particularly γδ T cells, also participate in this process, in both human [6,7,8] and animal models of PV [9,10,11].

Mature, human γδ T cells predominantly segregate into Vδ1 and Vδ2 subsets [12] that preferentially populate epithelial barriers and blood, respectively [6,13,14,15,16,17,18]. Both the Vδ2 and nonVδ2 subsets can be further divided into type 1- (cytotoxic, TBX21^+^EOMES^+^IFNG^+^) [19,20,21], type 3- (RORC^+^IL17A^+^IL18R1^+^) [22,23], and type 2-like (ZBTB16^+^) [24] effector cells. More recently, another classification scheme has been put forward based on the bimodal distribution of γδTCR surface expression in fluorescence-activated flow cytometry [23]. Two distinct classes of blood and tissue γδ T cells have thus been proposed: γδ^hi^ cells, which largely align with TRDV1 usage, and γδ^int^ cells, which adopt a more nuanced TRDV profile (at least in the bloodstream). The exact fractions, however, vary widely between different individuals and populations [23,25]. In addition, high-throughput RNASeq studies have identified many more distinct subsets of γδ T cells [19,22,26], but their biological significance for PV is virtually unknown. For example, numeric aberrations of γδTCR^int^ (blood), Vδ2^+^ γδTCR^int^ (blood), and (Vγ9)Vδ2^+^ T cells (lesional skin and blood) have been reported [6,25], but the exact mechanism that underpins those alterations is unknown. A challenge for future research will be to account for all the processes contributing to γδ T-cell granularity in human PV.

In this study, we examined the microRNA (miRNA) expression and its relation to the γδ^hi^:γδ^int^ dichotomy in the circulating γδ T cells of PV donors. Endogenously expressed miRNAs are well-established epigenetic regulators of T-cell development and function, with growing evidence demonstrating their critical role in various autoimmune diseases [27]. As such, PV has also been linked to the aberrant expression of >400 miRNAs [28], most of which have been identified in full-depth biopsies of involved and non-involved psoriatic skin [29,30,31,32,33,34,35]. Several whole blood [36] and exosome-derived [37] miRNAs have also been studied, showing potential as biomarkers and instruments for a PV diagnosis [38], prognostication [39], and developing epigenetic therapy [40]. Nonetheless, very few of those associations have so far been confirmed in PV, and even fewer have been examined in circulating γδ T cells [41]. That includes the members of the miR-17~92, miR-29, miR-25~92, and let-7 families, which make up a part of the characteristic miRNA signature in psoriatic plaques [28,29,30,31,32,33,34,35] and play roles in TCR-mediated signaling, cytokine production [42,43], keratinocyte biology [44], type I [45] and innate [46] immunity, and T-cell survival [47]. This paper focuses on four of these miRNAs (miR-20a, miR-29a, miR-92, and let-7c), which mediate translational repression by pairing with the 3′-untranslated region of target mRNAs [48]. First, we tested for their differential expression in sorted γδ blood T cells from healthy controls and PV, then matched these findings to the γδ^hi^:γδ^int^ and Vδ2:Vδ1 composition by using cytometric data from our recent and updated study [25]. In Section 2.3, we further related the differentially expressed miRNAs to the bulk expression of their putative mRNA targets (as indicated by TargetScan, miRDB, and TarBase tools, Supplementary Table S1) in γδ T cells, namely, *ZBTB16*, *RORC*, *RUNX3*, *TBX21*, *EOMES*, *IL18R1,* and *SELPLG*. The associated methodology and data reduction methods are described in Section 4.7. The final section discusses the implications of our results and future challenges.

## 2. Results

### 2.1. Subject Characteristics

The detailed structure of our sample is described in [25], but we have summarized the relevant points here. The baseline characteristics of the subjects are shown in Table 1. No difference in the studied properties was observed between the two groups (PV vs. controls, Table 1). Most participants were mildly affected, young and middle-aged white males with a history of prior CMV infection, low systemic inflammatory burden, and normal-to-overweight score on the BMI scale. Both groups were well-balanced on the CMV status, a factor that strongly imprints on the γδ T-cell composition [49,50,51]. Neither the CMV viral load (DNAemia) nor the CMV glycoprotein-specific IgG binding were examined.

### 2.2. The γδ T-Cell Composition Is Reshaped by the CMV Infection History and the Case–Control Status

**Figure 1:**
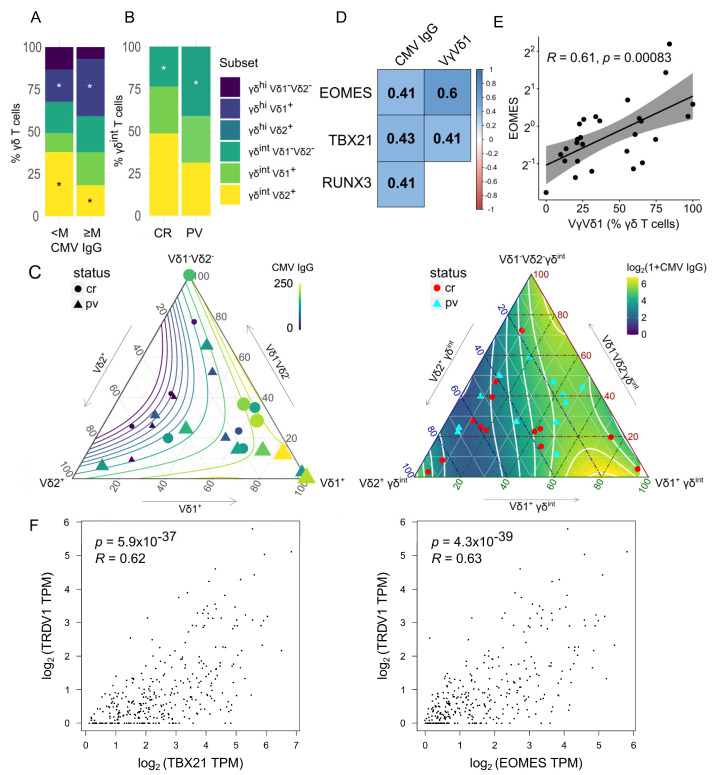
Stacked barplots (average percentage) showing the γδ blood T-cell composition by (**A**) cytomegalovirus (M corresponds to median serum CMV IgG, 132 IU/mL, panel A, 12 + 12 donors) and (**B**) case (PV, *n* = 13)–control (CR, *n* = 14) status. An asterisk denotes a statistically significant difference according to the two-tailed Mann–Whitney test, * *p* < 0.05. Only significant differences are plotted. (**C**) The distribution of γδ T-cell subsets in ternary coordinates. The three coordinates add to a constant of 100%. The color-coded isocontour lines of serum CMV IgG levels have been smoothened to filter out local features. Left panel: The color and size of the points are determined by the value of the serum CMV IgG (color bar). Right: The background shows color-coded CMV IgG levels. (**D**) Gene expression associations with the γδ blood T-cell composition and serum IgG levels. The colored scale bar reports Spearman rank correlations (R). All correlations are significant at *p* < 0.05. (**E**) Scatter plot representing a relation between the *EOMES* gene expression in bulk γδ blood T cells and their composition (Vδ1^+^ fraction, flow cytometry). R denotes the Spearman’s correlation coefficient; each dot represents one donor. The black line represents a linear model fit (least squares method), whereas the shaded region indicates the 95% confidence interval. The Y-axis (fold change) is log-scaled. (**F**) Expression levels of the *TRDV1*, *EOMES,* and *TBX21* genes in the whole blood samples [*n* = 337, log-transformed (1 + TPM) values, GTEx project, USCS Xena, gepia2.cancer-pku.cn]. Each dot represents one donor. Numbers denote Spearman’s correlation coefficients and their *p*-values. TPM transcript per million.

As previously shown [25], the γδ T-cell composition was heavily influenced by the CMV infection history (Figure 1A) and the case–control status (Figure 1B). To illustrate this behavior, we divided the participants by the median CMV IgG level, irrespectively of case–control data. The total number of γδ T cells was similar in both CMV IgG groups, but their composition changed (Figure 1A, Source Data). As expected, the PB Vδ1^+^ γδ cells were numerically expanded in the highly CMV-experienced environment [49], replacing the γδ^int^ Vδ2^+^ populations (Figure 1C). At the transcriptional level, *EOMES*, *TBX21,* and *RUNX3* expressions were much stronger at the higher end of the IgG CMV range (Figure 1D), culminating in the highest Vδ1 cell densities (Figure 1D,E, Supplementary Figure S2A,B,D–F), consistent with their transition to the cytotoxic/effector program [19,26,52] (Supplementary Figure S2B,D,F, Supplementary Figure S3). This agrees with the observational results from the independent bulk (Figure 1F) and two scRNASeq studies [26,53]. As expected for a middle-income country [54,55,56], a high prevalence of CMV seropositivity was observed, which could help explain a higher proportion of TRDV1 usage in γδ^hi^ cells from our cohort [25] compared to some other populations [23]. This suggests that even more complex effects of the CMV on γδ T-cell biology may be expected [50,51,57]. Still, a small, subdominant channel of γδ cell remodeling would be challenging to detect with the current sample size. In addition, a loosely constrained proxy parameter such as CMV IgG may not possess the desirable properties for this task [58]. As a result, we chose to rigorously account for any potential bias stemming from CMV exposure in our downstream analysis.

In contrast to the CMV, Figure 1B shows that in PV γδ^int^ Vδ1^-^Vδ2^-^ cells, a poorly characterized subset of clonally divergent γδ T cells was found in relative excess. Again, no change in total γδ T-cell frequencies was found (Source Data). The reason for this excess of Vδ1^-^Vδ^2-^ cells in the PV γδ^int^ fraction remains to be explained. No change was observed in other cell subsets (Source Data).

### 2.3. The Differential Expression of miR-20a Is Associated with Transcriptional Variations in Bulk γδ T Cells and the Altered Vδ Composition of the γδ^int^ Compartment

**Figure 2:**
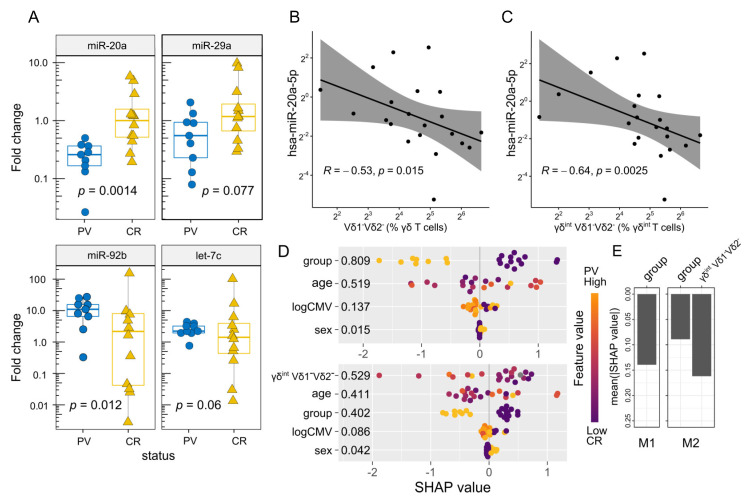
Box–whisker plots (**A**) highlighting the miRNA expression (fold change, log2 scale) in each donor (FACS-sorted γδ blood T cells). Horizontal lines represent the median with the interquartile range. *p*-values are from a two-tailed Mann–Whitney U-test. Scatter plot representing (**B**) a relation between the hsa-miR-20a-5p expression (MIMAT0000075, fold change, log2 scale) and (**C**) the γδ blood T-cell composition (flow cytometry). R denotes the Spearman’s correlation coefficient; each dot represents one donor. The black line represents a linear model fit (least squares method), whereas the shaded region indicates the 95% confidence interval. Each dot represents one donor. The (**D**) hsa-miR-20a-5p expression in bulk γδ blood T cells, multivariate assessment (fold change, log scale). The estimates in the figure compare the predictions obtained with and without γδint cell composition in the model (lower vs. upper panel). The beeswarm plots display Shapley values (SHAP) per feature using min.-max. scaled feature values on the color axis. The color of the points represents the value of the feature from low to high (i.e., higher feature values are redder), providing information about the direction of the association between the predictor and the miRNA levels. Numbers represent the average absolute Shapley value per predictor: The larger the absolute SHAP value, the greater importance of the predictor for the model’s output. Each dot represents one donor. The logCMV = log2(1 + CMV IgG) group corresponds to the case–control status. (**E**) Predictive importance of the case–control status (group) with (M2) and without (M1) cell composition (TRDV usage, % γδint T cells) for the hsa-miR-20a-5p expression. Mean absolute SHAP values, computed according to Wodtke et al. (https://github.com/gtwodtke/nhood_mediation_airToxics [60], accessed on 6 December 2022). Each fit includes baseline variables (age, sex) and covariates (CMV IgG, body mass index).

**Figure 3:**
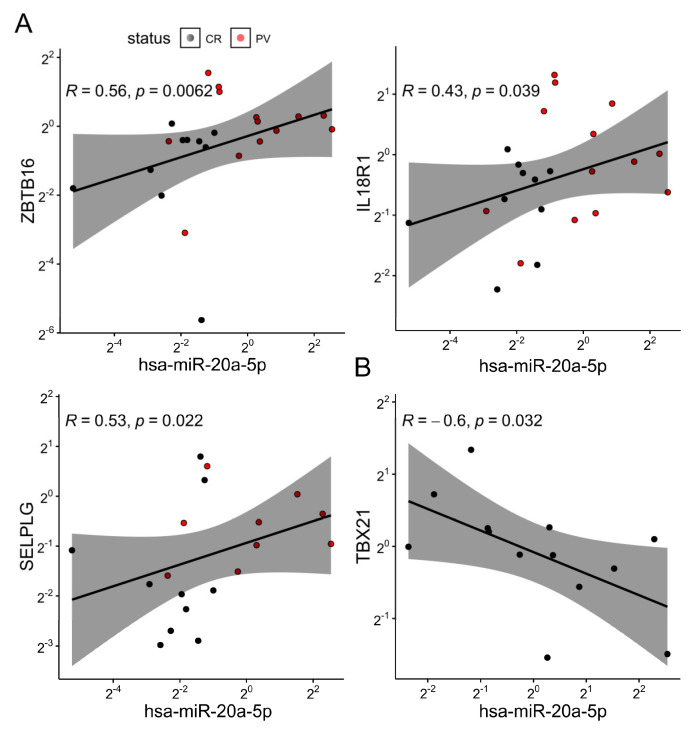
(**A**) Scatter plot representing relations between the hsa-miR-20a-5p expression and some of the gene markers used in the pooled sample (bulk γδ blood T cells, fold change, ΔΔCt method). Dots are colored according to their group membership (controls/CR black, cases/PV red). (**B**) Scatter plot representing the association between the hsa-miR-20a-5p and the *TBX21* expression in controls (*n* = 13). Both axes are log-scaled. R denotes the Spearman’s correlation coefficient; each dot represents one donor. The black line represents a linear model fit (least squares method), whereas the shaded region indicates the 95% confidence interval.

Next, we investigated the effect of PV on selected peripheral blood γδ T-cell miRNAs. For this purpose, we considered several poorly studied miRNA candidates (miR-20a, miR-29, miR-92b, and let-7c), which potentially target *RORC*, *RUNX3*, *TBX21*, *EOMES*, *IL18R1, ZBTB16,* and *SELPLG* transcripts encoding proteins instrumental in γδ T-cell commitment and differentiation (Supplementary Table S1). The results showed that the bulk miR-20a values were lower in PV compared to control γδ T cells (Figure 2A). Apparently, this loss of miR-20a expression was largely (but not completely) dependent on increasing Vδ1^-^Vδ2^-^ (Figure 2B), and particularly, γδ^int^ Vδ1^-^Vδ2^-^ cell densities (Figure 2C,D), indicating different timescales for their production (Vδ1^-^Vδ2^-^ cells vs. miR-20a). This is potentially explained by the suppressed miR-20a formation in certain Vδ1^-^Vδ2^-^ lineages, which may lead to less efficient miR-20a enrichment. The inclusion of age, sex, and CMV status in the model did not materially affect these results (Figure 2D,E). Notably, even though PV and miR-20a were both associated with Vδ1^-^Vδ2^-^ γδ T-cell numbers, these relations need not be mediated by the same Vδ1^-^Vδ2^-^ subset. The miR-20a levels were also decoupled from any other studied cell types and mRNA levels of their predicted targets (*RUNX3* and *RORC*), but coincided well with the *ZBTB16*, and to a lesser degree with the *IL18R1* and *SELPLG* expressions (Figure 3A), which, in turn, were predominantly associated with TRDV2 usage (but not vice versa; *ZBTB16*, *IL18R1*, Supplementary Figure S2G,H), innate-like differentiation (*ZBTB16*, Supplementary Figure S2D,E), and cell trafficking (*SELPLG*, Supplementary Figure S3). No statistical evidence of inverse miRNA–mRNA association was found in the pooled analysis, although it has been shown that miRNAs can decrease their target availability. This, however, is highly model-dependent, as the relationship relies heavily on the strength of miRNA–mRNA coupling. In complex cellular mixtures, m(i)RNA composition is primarily determined by cell lineage, the proportion of each cell type, and cell-type-specific programs. miRNAs, by contrast, impart complex [59], mostly weak negative effects on their targets. Under such conditions, modest relations (if any) may be missed, so their absence in leading-order approximations may not be surprising. Nevertheless, a weak reciprocal relation between the miR-20a and the *TBX21* expression was observed in the control samples (Figure 3B). This, however, neither confirms a mechanistic relationship nor precludes effects at the protein level.

We also detected a difference in miR-92b abundance between the two groups (13.2-fold enrichment, PV vs. controls, bulk γδ T cells, Figure 2A), but it lacked association with either cell numbers, levels of putative target mRNAs (*ZBTB16* and *SELPLG*), or any other biological covariate. For miR-29a and let-7c, no evidence of differential expression by case–control status was observed nor association with the expression of predicted target genes (*TBX21, EOMES,* and *ZBTB16*, respectively). The miR-29a expression was higher in men than in women (Source Data); however, this statement must be toned down due to the small number of female participants. Altogether, these results point to the role of the miR-20a in regulating the compositional and transcriptional features of the γδ T-cell pool in PV. The results, however, rest largely on mild illness, raising the question of their applicability to more evolved disease settings. Therefore, much remains unknown about miRNA distribution under realistic conditions.

## 3. Discussion

The γδ T cells play an essential role in animal models of the disease [7,9,10]. However, drawing a direct connection between the properties of human γδ T cells and their murine counterparts is not possible. As a result, a mechanistically relevant population has yet to be identified among many different γδ T-cell populations. 

Here, the subsets at hand are the γδ^hi^/^int^ cells, a largely neglected category that owes its name to a distinct pattern of γδTCR surface expression in flow cytometry. Current evidence suggests that γδ^hi^ cells differ from γδ^int^ cells by the effector cytokines they produce, TRDV usage, and key transcription factors [23,25]. This notion is further underscored by preclinical data, suggesting that γδ^hi^ cells are selectively associated with synovial inflammation in patients diagnosed with spondyloarthritis, a common companion to psoriasis [60].

We improved upon the existing literature in several ways. First, we demonstrated that PV can be sufficient to increase the relative size of the nonVδ1nonVδ2γδ^int^ compartment in the bloodstream, although no evidence exists that this is enough to change the composition of skin T cells. This updates the result from Plužarić [25]. Second, a significant downregulation of the miR-20a was observed in PV patients. We found that bulk γδ T cells, depleted from the miR-20a host’s larger Vδ1^-^Vδ2^-^γδ^int^ population in the blood, lose reciprocally larger amounts of transcripts commonly associated with Vδ2 cells [22,25], such as those encoding DNA-binding factors (*ZBTB16*), cytokine receptors (*IL18R1*), and cell adhesion molecules (*SELPLG*) [22,61], while the levels of miR-20a-predicted targets, related to cytotoxic effector (*RUNX3*) and Th17-like (*RORC*) γδ T-cell subsets, were apparently not affected [22]. The total number of γδ T cells did not change in the process, indicating a change in composition, rather than in the size of the circulating γδ T-cell pool. Although we cannot definitively assign this result to a single biological process, these findings are broadly consistent with the suggested blood-to-skin trafficking of Vγ9Vδ2 cells in PV [6], and with reports of a diminished miR-20a expression in joint-infiltrating Vγ9Vδ2 T cells from rheumatoid arthritis [62]. Correspondingly, an increased miR-20a-5p expression has been repeatedly observed in normal-looking and affected human psoriatic skin [28,31]. In terms of functioning, a lower miR-20a expression was previously associated with stronger TCR-mediated signaling and cytokine secretion in CD4^+^ T cells [42], as well as with improved NK cell-killing capacity [63]. Similarly, mice lacking miR-17~92 in mature CD8^+^T cells exhibit enhanced memory differentiation and lymphoid homing of T-bet^lo^CD8^+^T cells upon the LCMV challenge [64]. Additional miRNAs that might underlie changes in peripheral γδT cell composition in PV remain to be addressed, as case–control differences in miR-29a and let-7c expression were imperceptible, at least at the level of bulk γδ T-cell transcriptome. This calls for a deeper analysis of miRNAs and their interplay with distinct γδ T-cell populations in PV. Meanwhile, the mechanisms leading to Vδ1^-^Vδ2^-^γδ^int^ cell accumulation in the blood are still unknown.

The case of miR-92b overexpression in bulk γδ T cells is more enigmatic, given the absence of any cellular context or mRNA relationship. Higher levels of miR-92b-5p and its antisense pair, miR-92b-3p, have been observed in non-lesional skin [28,31] and psoriatic keratinocytes [32], respectively, supporting the role of this miRNA family in PV. Elevated miR-92b levels were also reported in activated T cells, and implicated in the negative feedback regulation of the calcineurin/NFAT signaling pathway [65]. Unfortunately, very few studies specifically analyzed the impact of miRNAs on the γδ T-cell properties.

There are also a few limitations in our study that should not be overlooked. First, the γδ^hi^/^int^ cells have not been examined to the same depth and extent like the other T cells. This triggers a series of important questions: How are the γδ^hi^/^int^ cells related to the γδ T-cell clusters from high-throughput studies? How does this dichotomy translate into biological differences? Does the clonotypic composition of Vδ1^-^Vδ2^-^γδ^int^ cells change in PV? This, however, is beyond the scope of the present study and an attempt at a unified description must be left to future studies. Second, the γδ^hi^/^int^ cells have been dichotomized according to the fluorescence intensity of the cells stained with the pan-γδTCR antibody. This dichotomy, however, is far from perfect. A more diffuse pattern of staining can be observed in some individuals [25,66]. In others, the γδ^hi^ and γδ^int^ populations of blood T cells may be split into multiple clusters [25]. Thus, methodological and technical variations between the studies may result in significant inconsistencies. In addition, genetic diversity, infection history, and environmental effects may conspire to obscure the results in human studies. To provide confidence in these new results, we performed a rigorous check against confounding by common covariates. Third, we used RNA from bulk γδ T cells, thus precluding an efficient probe into target mRNA silencing by miRNAs. This further emphasizes the importance of perturbative studies in highly resolved and carefully purified cell populations, avoiding cell mixtures. The complementary approach is to perform comprehensive (genome-wide) miRNA profiling, which is particularly important when considering spillover effects arising from tightly co-expressed miRNAs. Fourth, direct measurements of miRNA abundance suffer from sensitivity limits in low-expressing cell populations; therefore, they are likely biased towards targets and samples where such a measurement is possible, but are not representative of the population-level trend. As a result, some degree of incompleteness is generally expected at the lower end of the miRNA expression, which effectively puts the obtained estimates closer to their upper boundaries. Improved measurements will be necessary to resolve the existing uncertainties. Finally, mechanistic insight is central to the validity of these findings, posing an unmet need for a deeper, orthogonal characterization of miRNA biology in γδ^hi^/^int^ cells. This would help identify not only the most promising candidates, but also potential targets in PV that could be exploited for a prognostic or therapeutic effect.

Despite these imitations, our new analysis provides an updated insight into the γδ^hi/int^ partition of blood T cells and its association with the miRNA expression in PV. Elucidating the biological mechanism is essential for interpreting the data from our and future observations.

## 4. Materials and Methods

### 4.1. Study Design and Subject Selection

We used archival RNA samples extracted from flow-sorted CD3^+^γδTCR^+^ lymphocytes of 13 clinically active, therapeutically naïve psoriatic patients (PV) [(M/F ratio: 10/3; median years of age (IQR): 35(28–43)], and 14 sex- and age-matched, unrelated healthy controls [(M/F ratio: 9/5; 32(28–41) years of age); Table 1.]. Study participants were originally recruited at the Department of Dermatology and Venereology, University Hospital Center Osijek, following physical examination and pathohistological confirmation of psoriasis vulgaris. Disease severity and the impact on the quality of life were assessed using the PASI (Psoriasis Area and Severity Index) and the DLQI (Dermatological Life Quality Index) questionnaires, respectively. The serological markers of past bacterial (QuantiFERON-TB Gold test) and viral exposure (anti-CMV IgG, anti-CMV IgM, anti-HBsAg, anti-HCV) were tested at the time of recruitment, together with a complete blood count (CBC), C-reactive protein (CRP) serum levels, erythrocyte sedimentation rate (ESR), and body mass index (BMI). Patients on either systemic immunomodulation or cytostatic therapy, with malignant, autoimmune, and infectious diseases or allergic reactions within 6 weeks before diagnostic processing were excluded from the study. Written informed consent was collected from all participants prior to sample collection. The study protocol was reviewed and approved by the Ethics Committee of the Faculty of Medicine in Osijek (number: 2158-61-07-18-135).

### 4.2. The miRNA Selection

In order to select the miRNAs targeting previously tested mRNAs (*RUNX3, IL18R, ZBTB16, RORC, TBX21, EOMES*, and *SELPLG*) [25], we relied on reports of previously validated targets [40,41,42,43,44,45,60,61,62,63] and three target prediction algorithms, namely, TargetScanHuman (Release 8.0) [67], miRDB [68], and TarBase (v.8) [69]. These algorithms incorporate computational methods and experimental validation to predict miRNA–mRNA interactions and they have been widely used in the field of miRNA research [65,70,71,72,73]. In line with that, high target score predictions in at least one target algorithm, or simultaneous targeting of at least two tested mRNAs were used as the miRNA selection criteria (Supplementary Table S1). Some candidates were targeted by multiple miRNAs.

### 4.3. Isolation of Peripheral Blood Mononuclear Cells

Peripheral blood mononuclear cells (PBMCs) were isolated from 10 mL of freshly collected, heparinized blood samples and fractionated by density gradient centrifugation on the Lymphoprep medium (Stemcell Technologies, Vancouver, Canada), as advised in the manufacturer’s leaflet. In short, 10 mL of whole blood was diluted with saline (0.9% (*w*/*v*) NaCl) in the 1:1 ratio, carefully layered onto 15 mL of the Lymphoprep medium, and sedimented into leukocyte fractions during a 25 min centrifugation at 800× *g*, with break off. The harvested mononuclear cells were carefully washed twice in phosphate-buffered saline (PBS), followed by resuspension and 10 min centrifugation at 550× *g*. The PBMC numbers were determined with the use of the Countess II automated cell counter (Thermo Fisher Scientific, USA) and aliquoted for flow cytometry (min 1 × 10^6^ cells) and fluorescence-activated cell sorting or FACS (min. 6× 10^6^ cells).

### 4.4. Flow Cytometry Analysis and γδT Cell Sorting

The flow cytometry of peripheral blood γδT cells was accomplished by monoclonal antibody staining of CD3ϵ (FITC, 1:250, clone UCHT1 gamma, produced at the Department of Immunology and Biotechnology, University of Pecs), TCRγδ (PE-Cy7, 1:100, clone B1, BioLegend), TCRVδ1 (APC, 1:100, clone TS8.2, eBiosciences), and TCRVδ2 (PerCP/Cy5.5, 1:200, clone B6, BioLegend)] surface markers. Dead cells were excluded using LIVE /DEAD Fixable Near IR Dead fluorescent viability dye (ThermoFisher Scientific, Rockford, IL, USA) and unspecific antibody binding was prevented by a 10 min pre-staining incubation with the 5% FcR blocking reagent (TruStain FcX, Biolegend). The γδT cell-count acquisition was performed with the use of the BD FACS Canto II cytometer (FACS Canto II, Becton Dickinson, San Jose, CA, USA) and the collected data were analyzed with FlowLogic v7.2.1. software (Inivai Technologies, Mentone, VIC, Australia). The gating strategy for peripheral γδT cell populations (Supplementary Figure S1) was set according to compensation parameters selected by fluorescence-minus-one (FMO) and single-stained control processing, as described in more detail previously [25]. The second, larger aliquot of paired PBMC samples was used for cell sorting of CD3^+^γδTCR^+^ expressing cells on a 4-color S3e cell sorter (Bio-Rad Laboratories, Hercules, CA, USA). As previously reported, a minimum of 15,000 sorted γδT cells were collected from each freshly collected PBMC aliquot, directly into the miRVana™ miRNA Lysis/Binding buffer (Thermo Fisher Scientific, Rockford, IL, USA) and used for subsequent RNA extraction, which was done according to the manufacturer’s instructions. The purity of the sorted γδT cells was estimated using RNASeq analysis of the α-, β-, γ-, and δ-chain TCR repertoire (Archer Immunoverse High Sensitivity TCR Kit, Illumina MiniSeq sequencer, manuscript in preparation). The results of the TRA/TRB/TRG and TRD CDR3 clonotype analysis (Archer Analysis Software) for one representative sample are given in Supplementary Table S2.

### 4.5. cDNA Synthesis and mRNA/miRNA Expression Analysis

Before being processed into cDNA, cryopreserved total RNA samples were thawed and the available quantities were measured using the DeNovix QFX Fluorometer (DeNovix Inc., Wilmington, USA). Reverse transcription (RT) of four candidate (hsa-miR-20a-5p, hsa-miR-29a-3p, hsa-miR-92b-5p, hsa-let-7c-5p) and three control miRNAs (hsa-miR-192-5p, hsa-miR-345-5p, hsa-miR-423-3p) was carried out in four sequential steps using the TaqMan Advanced miRNA cDNA Synthesis kit (Thermo Fisher Scientific, Rockford, IL, USA). In short, for initial 3′ poly-A tailing, 5 ng of total RNA was incubated in 5 µL of Poly(A) reaction mixture (45 min at 37 °C) and the 5′ ligation of an adaptor sequence in a 15 µL ligation blend (60 min at 16 °C) was performed to extend all mature miRNAs present, prior to cDNA synthesis. Next, the extended miRNAs were reverse transcribed (15 min at 42 °C) in a 15 µL RT reaction mix composed of 6 µL of 5X RT buffer, 1.2 µL of dNTP mix, 1.5 µL of 20Xuniversal RT primers, 3 µL of 10X RT enzyme mix, and 3.3 µL of RNase-free water. In order to improve the detection of low-expressing miRNA targets while maintaining their relative differential expression levels, 5 µL of each cDNA sample was pre-amplified with 2.5 µL of Universal miR-Amp Primers and 25 µL of miR-Amp Master Mix. The pre-amplified cDNA products were diluted fivefold and the transcript levels of target miRNAs were measured using the QuantStudio 5 Real-Time instrument (Thermo Fisher Scientific, Rockford, IL, USA) in triplicate 15 µL quantitative real-time PCR (qRT-PCR) reactions containing 6.75 µL of the cDNA template, 7.5 µL of TaqMan Fast Advanced Master Mix, and 0.75 µL of TaqMan Advanced miRNA Assay (Applied Biosystems Foster City, CA, USA). The cycling conditions were set according to the guidelines in the manufacturer’s leaflet and the list of assays is given in Table 2.

The threshold cycle (Ct) values were collected using QuantStudio Design&Analysis software, v1.5.2. Amplification efficiency and pipetting precision, as assessed by the linear regression coefficient (R^2^), were measured by five-point, fourfold serial dilutions of the arbitrary standards that were run next to the samples in each experiment, providing an insight into the final achieved ranges of efficiency (80–100%) and R^2^ (0.980–0.998). Intra-assay variability was less than 1.96%, and a variation of less than 2.34% was achieved between different PCR experiments. Among the three tested control miRNAs, only the hsa-miR-423-3p was successfully amplified in our sample set and thus used for the NormFinder stability evaluation (M = 2.098) and normalization of target miRNAs expression levels. Finally, the fold difference in the relative miRNA quantity was determined with respect to the control group levels, using the efficiency corrected model of the 2^−ΔΔCt^ method as described by Pfaffl [74]. The observed differences in miRNA expression were analyzed relative to the previously collected data on the peripheral γδT cell phenotype, frequency, and transcriptional reprogramming, as well as changes in cytokine and chemokine serum levels of PV patients. The quantification of mRNA for *EOMES*, *RUNX3*, *TBX21*, *RORC*, *CCR6*, *ZBTB16*, *SELPLG*, and *IL18R* was performed as reported earlier [25].

### 4.6. In Silico Analysis

The processed single-cell (sc)RNASeq data from Tan et al. [22] were used for creating example figures. The dataset, including cell-type annotations, was downloaded from the Gene Expression Omnibus (GEO), accession number GSE149356 (FACS-sorted human γδ T cells from 2 cord blood donors and 2 adult blood donors, 10X Genomics). The analysis was carried out through Seurat v3.2.3 [52] and Nebulosa v1.6 [75] pipelines. For bulk RNAseq, 337 whole blood samples from the GTEx project [76] were processed using the Gene Expression Profiling Interactive Analysis interface [77] (http://gepia2.cancer-pku.cn, accessed on 20 August 2022). For this analysis, we restricted ourselves to gene–gene cross-correlations by adopting the harmonized TPM (transcript-per-million) data from UCSC Xena [78].

### 4.7. Statistical Analysis

Gaussianity was assessed by the Shapiro–Wilk test, and the homogeneity of variances by Levene’s test. Generally, a nonparametric approach was adopted. Where possible, an equal allocation design was used to maximize statistical power. Continuous data is presented as median with the interquartile range (IQR), except for stacked barplots, where arithmetic means were utilized, because the sum of group-level medians does not readily converge on the grand median. This choice did not significantly affect our results. For downstream analysis, serum CMV IgG levels were winsorized at the upper limit of quantification (250 IU/mL). As most subjects were CMV-experienced, we also explored the effect of past CMV exposure by dividing the sample into two equal subgroups using median CMV IgG quantity. The Mann–Whitney U-test was used for independent group comparisons and the Fisher’s exact test was applied to contingency tables. Pairwise correlations were assessed by the Spearman’s rank test. Shapley’s additive explanations [79], representing the Shapley value decomposition of a multivariate model, were used to determine feature importance, i.e., their marginal contributions to target variables [80]. The computed Shapley values perform reasonably well in sparse models, when predictors are moderately correlated (shapviz v0.2.0 package). Baseline covariates (age, sex, CMV IgG) and the case–control status were used as predictors affecting miRNA expression. The SHAP values were then obtained by fitting the model with and without the cell composition included as a predictor. This allowed us to identify which covariates are likely to play a more vs. less important role in shaping miRNA expression. We also modeled a relationship between putative mediators (case–control status, cell composition) and the miRNA expression by adjusting for a set of baseline features (age, sex) and covariates (CMV IgG, BMI). To this end, we adopted a recently developed framework that can accurately handle interactions and nonlinearity, while minimizing problems due to overfitting [81]. The overall result did not differ qualitatively between the two approaches. For ternary diagrams, color maps were interpolated by fitting 2nd- and 3rd-order polynomials in Cartesian space under the general linear model. Each contour fit (isovalue line of a quantity) was checked for accuracy and consistency. Where appropriate, log-transformed data were used.

If not otherwise stated, a two-tailed *p* < 0.05 was considered significant. No adjustment for multiple testing was applied. All statistical analyses were performed in R v4.0.3 (R Core Team, www.r-project.org). The boxplots, barplots, scatterplots, and ternary maps were generated using R-package cowplot v1.1.0, ggplot v3.3.5, ggpubr v0.4.0, ggtern v3.3.5, patchwork v1.1.1, RColorBrewer v1.1.2, reshape2 v1.4.4, rstatix v0.7.0, scales v1.1.1, Ternary v2.1.0, tidyverse v1.3.0, viridis v0.5.1, viridisLite v0.3.0, and xgboost v1.3.2.1.

## Figures and Tables

**Table 1:** Study participants.

Group	PV	Controls	*p* *
N (male/female)	13 (10/3)	14 (9/5)	0.678 **
Age (chronological, years)	35 (28–43)	32 (28–41)	0.528
PASI	6.8 (5.5–11.5)	–	–
DLQI	3 (0.5–6.5)	–	–
BMI (kg/m^2^)	26.5 (21.3–30.1)	23.9 (20–25.7)	0.055
CRP (mg/L)	2 (0.7–2.8)	0.7 (0.3–1.7)	0.068
Anti—CMV IgG (AU/mL)	130 (19–178)	135 (42–171)	0.86
Anti—CMV IgG (pos/neg)	8/2	11/3	1 **

Continuous data are shown as the median (interquartile range). BMI—body mass index, CRP—C reactive protein, CMV—cytomegalovirus, PASI—Psoriasis Area and Severity Index, DLQI—Dermatology Life Quality Index, PV—psoriasis vulgaris, IgG—immunoglobulin G. * *p*-value, Mann–Whitney U-test. ** Fisher’s exact test.

**Table 2:** List of TaqMan assays for miRNA analysis.

Assay ID	miRBase ID	miRBase Accession Number	Mature miRNA Sequence
478586_mir	hsa-miR-20a-5p	MIMAT0000075	UAAAGUGCUUAUAGUGCAGGUAG
478587_mir	hsa-miR-29a-3p	MIMAT0000086	UAGCACCAUCUGAAAUCGGUUA
478577_mir	hsa-let-7c-5p	MIMAT0000064	UGAGGUAGUAGGUUGUAUGGUU
479207_mir	hsa-miR-92b-5p	MIMAT0004792	AGGGACGGGACGCGGUGCAGUG
478327_mir	hsa-miR-423-3p	MIMAT0001340	AGCUCGGUCUGAGGCCCCUCAGU

## Supplementary Materials

The following supporting information can be downloaded at: https://www.mdpi.com/article/10.3390/ijms24054323/s1.
