# Supplementary material for: The miR-20a/miR-92b Profile Is Associated with Circulating γδ T-Cell Perturbations in Mild Psoriasis
Source: Int J Mol Sci. 2023 Feb 21;24(5):4323. doi: 10.3390/ijms24054323 (PMC10001743; doi:10.3390/ijms24054323)
Supplement: Supplementary file 1 [file ijms-24-04323-s001.zip › Supplementary figures.pdf]

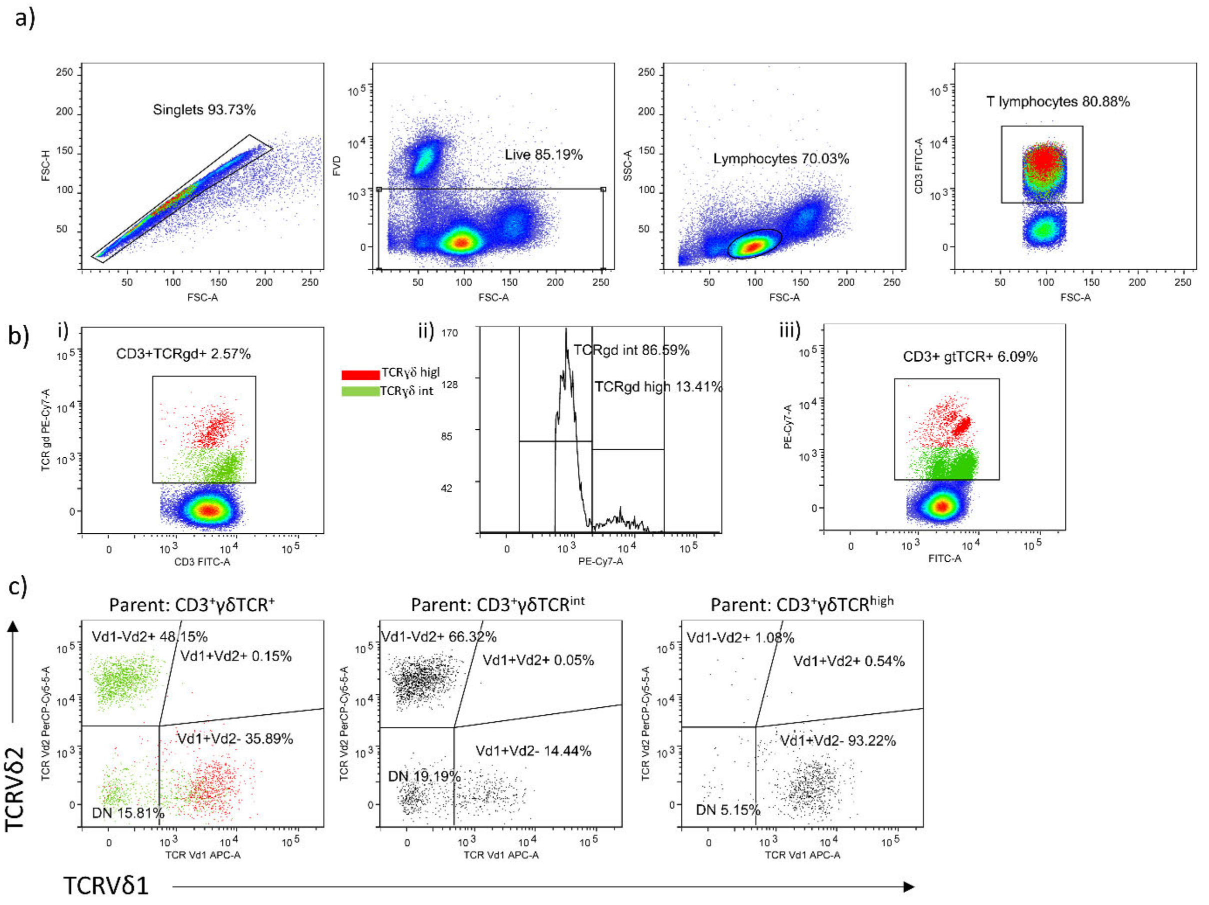

Supplementary Figure S1. Gating strategy of peripheral γδ T cells. (A) Representative dot plots illustrate the gating strategy for the identification of live, CD3+ T cells, (B) including gating of $CD3^{+}γδTCR^{int}$ and $CD3^{+}γδTCR^{high}$ and (C) $Vδ1^{+}$, $Vδ2^{+}$ and $Vδ1^{-}δ2^{-}$ T cell subsets analyzed within each $CD3^{+}γδTCR^{int}$ and $CD3^{+}γδTCR^{high}$ T cell panel. The $Vδ1^{+}δ2^{+}$ T-cell events were not further evaluated.

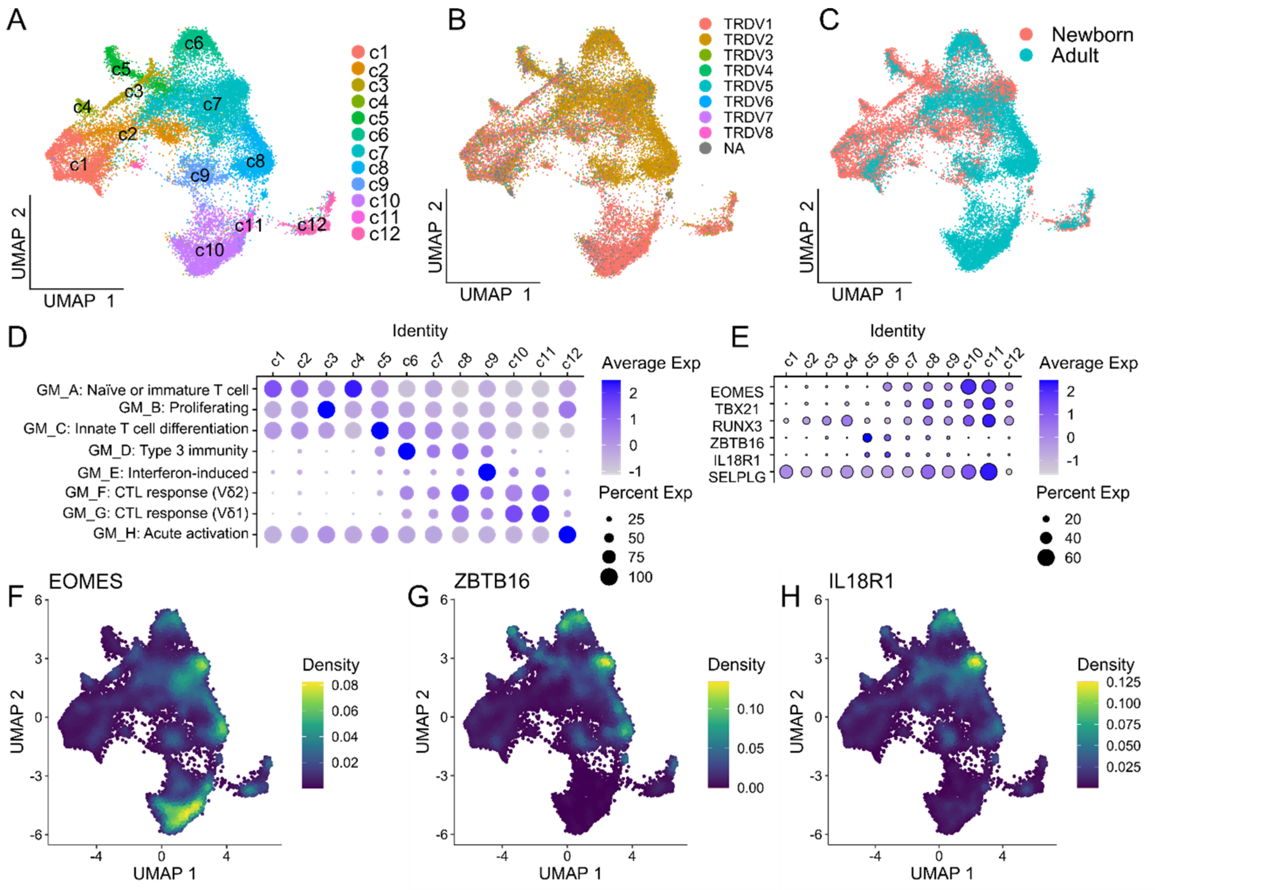

Supplementary Figure S2. UMAP (Uniform Manifold Approximation and Projection) visualization (25,904 cells) of the integrated scRNAseq data derived from the four 10X runs of TCRγδ⁺ T cells [21]. This yielded 12 distinct clusters (A) colored by TRDV usage (panel B, NA denotes cells without a TRDV gene). Fetal (cord blood) and adult cells are highlighted (C). Dot plots showing the expression of selected gene modules (GM_A:H, panel D, see Tan et al.) and markers (E) across the clusters. Dot size indicates the percentage of cells in each cluster expressing the gene, while color saturation indicates the average expression level of the cluster. F-H) Feature plots (weighted kernel density estimates) of some markers used.

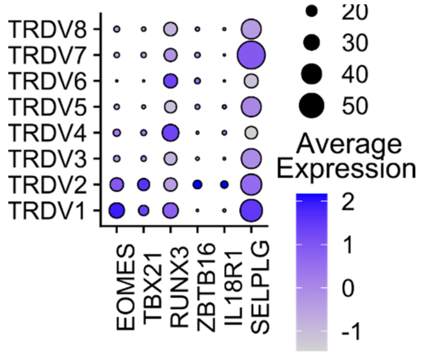

Supplementary Figure S3. Dot plots showing expression of selected transcripts according to TRDV usage (scRNAseq data, TCRγδ⁺ T cells from Tan *et al.*). Dot size indicates the percentage of cells in each cluster expressing the gene, while color saturation indicates the average expression level of the cluster.
