# Supplementary material for: The miR-20a/miR-92b Profile Is Associated with Circulating γδ T-Cell Perturbations in Mild Psoriasis
Source: Int J Mol Sci. 2023 Feb 21;24(5):4323. doi: 10.3390/ijms24054323 (PMC10001743; doi:10.3390/ijms24054323)
Supplement: Supplementary file 1 [file ijms-24-04323-s001.zip › Supplementary tables S1&S2.pdf]

Supplementary Table S1. miRNA predicted targets

miRNA	Target Scan			miRDB			TarBase		
	No. of predicted targets	Tested targets	Context score percentile	No. of predicted targets	Tested targets	Target Score	No. of predicted targets	Tested targets	Predicted Score
**hsa-miR-20a-5p**	*112*	*RORC*	*97*	*1381*	*RORC*, *RUNX3*	*94, 92*	*2909*	*RORC*, *RUNX3*	*0.919, 0.900*
**hsa-miR-29a-3p**	*1256*	*TBX21, EOMES*	*92, 93*	*1035*	*EOMES, IL18R1*	*85, 52*	*1691*	*SELPLG*	-
**hsa-miR-92b-5p**	*1086*	*ZBTB16, SELPLG*	*48, 58*	52	-	-	*31*	-	
**hsa-let-7c-5p**	*11*	*ZBTB16*	*73*	*990*	*RORC*	*61*	*2051*	-	
**hsa-miR-423-3p**	18	-	-	30	-	-	*414*	-	

Supplementary Table S2. Representative results of RNAseq TCR Profiling from flow-sorted γδ T cell sample

**HRZZ-19**				
No. of clonotypes total	4895			
Sequencing reads total	60565			
	**TRA**	**TRB**	**TRG**	**TRD**
No. of clonotypes	88	400	2850	1557
No. of clonotypes (TRA/TRB|TRG/TRD)	488		4407	
Clonotypes (of total, %)	1.8	8.17	58.22	31.81
Sequencing reads	248	2780	44364	13173
Sequencing reads (of total, %)	0.41	4.59	73.25	21.75
Sequencing reads (of total, %)	5		95	

* input=50 ng RNA
